# Polarization Sensitive Photodetectors Based on Two-Dimensional WSe_2_

**DOI:** 10.3390/nano12111854

**Published:** 2022-05-29

**Authors:** Andrey Guskov, Sergey Lavrov, Rinat Galiev

**Affiliations:** 1 Department of Nanoelectronics, MIREA—Russian Technological University, 119454 Moscow, Russia; guskov@mirea.ru; 2 V.G. Mokerov Institute of Ultra High Frequency Semiconductor Electronics of RAS, 117105 Moscow, Russia; rgaliev@isvch.ru

**Keywords:** 2D semiconductors, transition metal dichalcogenides, photodetectors, surface plasmon resonance

## Abstract

In this work we show the possibility of imparting polarization-sensitive properties to two-dimensional films of graphene-like semiconductors, using WSe_2_ as an example, by the application of ordered silver triangular nanoprisms. In addition, such nanoprisms made it possible to increase the optical sensitivity of optical detectors created on two-dimensional films by a factor of five due to surface plasmon resonance. The peculiarities of the surface plasmon resonance were shown by theoretical modeling, and the optimal conditions of its occurrence were determined. This article demonstrates an effective approach to creating spectrally selective, polarization-sensitive detectors based on two-dimensional graphene-like semiconductors.

## 1. Introduction

The main physical parameters of optical radiation are amplitude, polarization, frequency, and phase. Most optical sensors allow detection of light intensity only and are not sensitive to its polarization. The ability to analyze the optical polarization of the detected light greatly expands the capabilities of numerous applications, such as polarization-encoded quantum key distribution [1], biomedical applications [2], remote optical sensing techniques, object recognition [3], and polarization optical mapping [4].

For use in these applications, detectors must have high quantum efficiency and, at the same time, be compact enough to achieve a high level of integration and flexibility. To date, commercially available devices do not meet these requirements. To achieve polarization sensitivity, additional non-integrable polarizing filters or polarizers are installed directly in front of the optical detector, which significantly increases their size. In addition, flexible integrated devices are better integrated into optical systems, while classical photodetectors, due to their large size, are not suitable for flexible applications. The only possible approach to ensure the above requirements is the transition to new materials.

Among such new materials suitable for creating optical sensors, nanomaterials show the greatest promise. Some of them have significant optical anisotropy due to their shape, which allows selective detection of polarization. Such materials include nanowires [5] and carbon nanotubes [6], which are composed into ordered clusters and used as an active, flexible light-sensitive element. However, the creation of such structures is excessively time-consuming, because complex technological techniques are needed for their structuring and ordering.

Another approach is to use two-dimensional (2D) semiconductor films. These films, due to their unique quantum-dimensional effects, allow the creation of highly sensitive photodetectors [7] and sensors [8]. 2D semiconductors, such as transition metal dichalcogenides, were first obtained a relatively short time after the discovery of graphene in 2004. Since then, numerous attempts have been made to create optical detectors based on them. Some of these materials, such as ReSe_2_, have their own optical anisotropy due to the nature of their crystallographic structure. Thus, a polarization-sensitive photodetector was designed that allowed the detection of two polarization components independently of each other [9]. The only drawback of the above device is a not very high polarization selectivity, which limits the possibility of its application.

An alternative to the use of 2D materials that do not have sufficient intrinsic optical anisotropy is to create anisotropy using plasmonic effects. For example, the possibility of using elliptical plasmonic structures to achieve polarization sensitivity through local surface plasmon resonance (LSPR) was shown [10]. In this case, the 2D material used did not possess optical anisotropy at all, but when using gold structures, it became polarization sensitive. However, in this work, gold structures were used, which reduces the efficiency of such materials. Most importantly, the possibility of only local plasmon resonance near the metal/dielectric structure was taken into account, and not the surface resonance. At the same time, surface plasmon resonance (SPR) theoretically allows the increase of optical absorption up to 100%. Until now the possibility of SPR in such structures had not been investigated.

Different theoretical modeling approaches can be used to describe such structures. For example, it is shown in [11] that upgraded local density or generalized gradient approximations to density functional theory can be successfully applied to estimate the optical parameters of nanoscale hybrid metal–silicon materials. Moreover, for example, it was successfully demonstrated in [12] that ab initio molecular dynamics simulation approaches can be used to calculate the parameters of 2D graphitic-like aluminum nitride materials. Finite element modeling is another possible option for evaluating the optical properties of such complex three-dimensional systems, which is most widely used to estimate the effects of plasmonic amplification [13].

In this work, we investigated the possibility of creating highly efficient polarization-sensitive detectors by using SPR. For this purpose, arrays of triangular nanoprisms were created on the surface of the WSe_2_ monolayer film, which also made it possible to enhance the photosensitivity of the created photodetectors. To study the peculiarities of the surface plasmon resonance emergence, theoretical modeling of optical absorption for the created structures was performed, and the regularities of optical anisotropic absorption were determined.

## 2. Materials and Methods

There are many possible variations of the form factors of plasmonic structures designed to enhance the absorption of optical radiation in their adjacent materials by LSPR. To date, the effectiveness of spheres [14], ordered nanodiscs [15], PbS [16] and Ag [17] quantum dots, nanocubes [18], spherical dimers, nanostars, nanorods [14], grid arrays [19], and complex-shaped structures [20] has been demonstrated. However, only asymmetric nanostructures can have optical anisotropy, which significantly narrows their list of possible forms. For example, quantum dots, spheres, nanodiscs, or similar symmetric structures cannot be used for this purpose.

In such low-dimensional structures there are three main mechanisms that can be involved in increasing the absorption in the semiconductor layer, which is described in great detail in [21].

The first of them is the scattering of optical radiation on plasmonic elements. Usually, this mechanism plays a decisive role when the radiation scattered in this way further passes through the bulk layer of the semiconductor, being absorbed in it. Thus, scattering is required simply to increase the path length of the electromagnetic wave in the semiconductor. However, in this case, this effect makes almost no contribution. This is due to the fact that the plasmonic elements are located above the semiconductor, and most of the scattered radiation escapes into the air medium above. Moreover, the small thickness of the semiconductor, even if the scattered radiation reaches its surface, does not allow it to be effectively absorbed.

The second is the local surface plasmon resonance, which manifests itself in the collective oscillation of the electron density near the metal–semiconductor boundary. In the film itself there is a significant increase in the intensity of the electromagnetic wave, which in turn leads to an increase in the photocurrent. This effect is mainly dominant and allows one to obtain an increase in optical absorption using almost any form-factor of plasmonic structures on the surface of the semiconductor as shown in our work. The third mechanism is the surface plasmon resonance, which occurs mainly along long metal films, which also leads to redistribution of the electromagnetic field in the structures adjacent to it. It is worth noting that, in our case, only the latter two mechanisms yield contributions.

Therefore, the next factor in selecting the shape of the nanostructures is the effective generation of local surface plasmon resonance. For this purpose, such structures should possess sharp angles, which will enhance the electromagnetic field in their immediate vicinity. On this basis, the most effective form should have multipoint stars such as those mentioned in [14]. However, the characteristic sizes of such structures should be comparable to the length of the incident electromagnetic wave, which leads to technological difficulties in creating such defect-free structures with strictly specified geometric parameters, especially if they have a three-dimensional shape. Moreover, such multi-beam structures, for obvious reasons, begin to lose their anisotropic properties when the number of beams increases.

The next important factor is the mutual arrangement of such structures: if they are disordered, such as nanotubes lying on a substrate, then individually they will have optical anisotropy, but in the cumulative arrangement, this will be absent. For this reason, it is necessary to use asymmetric plasmonic structures, the arrangement of which relative to each other must be strictly ordered. The structures must be located as close to each other as possible. This is necessary because total amplification over the entire surface area of the functional material is more important than the amplification value of a single plasmonic structure.

The most effective material for the plasmonic element is silver, as it is extremely efficient at generating standing plasmonic waves [22]. So, for example, a comparison of metals used to create plasmonic materials is described in detail in [23]. In this work it is shown that the quality factor for localized surface plasmon resonances (at the 635 nm wavelength) in silver is two times higher than in gold. That is why in our work silver was chosen as a material for plasmonic structures. We also performed theoretical simulations for absorption in a 2D semiconductor film for different compositions of plasmonic structures (see Supplementary Materials S1). Thus, the simulation shows that the absorption in the TMD film is higher by 30% when using Ag rather than Au. It was also found that the choice of material of plasmonic elements does not affect their anisotropic absorption.

**Figure 1:**
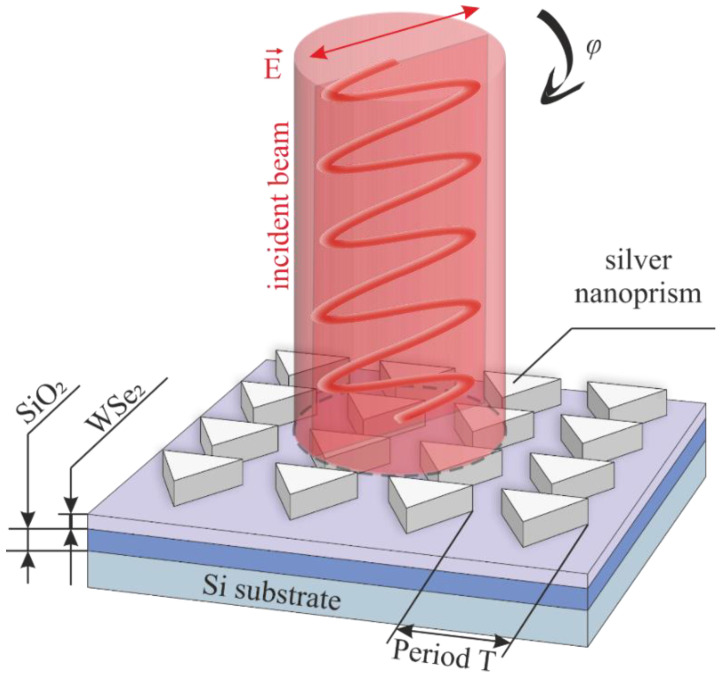
Schematic representation of an ordered triangular silver nanoprism on the surface of a 2D WSe_2_ film.

That is why the ordered plasmonic structures in the form of triangular silver nanoprisms were chosen in this work. Such nanoprisms are optimal candidates for optical amplification, as they fully satisfy all the above parameters. The prisms were arranged relative to each other in the form of a square rectangular matrix. This ordering of elements is usually the most commonly used for such problems. More so, it is optimal when creating detectors sensitive to polarization due to its symmetry. Schematic representation of a photodetector based on such a plasmonic structure is shown in Figure 1.

The distance between the elements of the matrix will hereafter be referred to as the period T of the plasmonic structures. The characteristic size of the plasmon element itself was chosen as a circle circumscribed around its triangular base. We considered it important to maintain the ratio of the open area of the TMD surface to the surface occupied by the plasmonic structures. Thus, we exclude the possibility of changing the efficiency of photodetectors only by changing the active area of the light-irradiated semiconductor. Three sets of plasmonic structures that have different periods were created: 200, 300, and 500 nm. A detailed description of the choice of the form factor of plasmonic structures is presented in Supplementary Materials S2. Thus, the optimal thickness of the plasmonic structures was estimated by simulation and the most efficient geometrical parameters were selected.

Standard silicon wafers with an oxide layer 90 nm thick were used as substrates for photodetectors, and two-dimensional WSe_2_ films were deposited on their surface by CVD method. To confirm the absence of defects in the semiconductor films and its homogeneity, standard techniques of luminescence microscopy, AFM, SEM, and nonlinear optical microscopy were employed. The standard technological route of electron-beam lithography was used to create ordered plasmonic structures with the required parameters.

**Figure 2:**
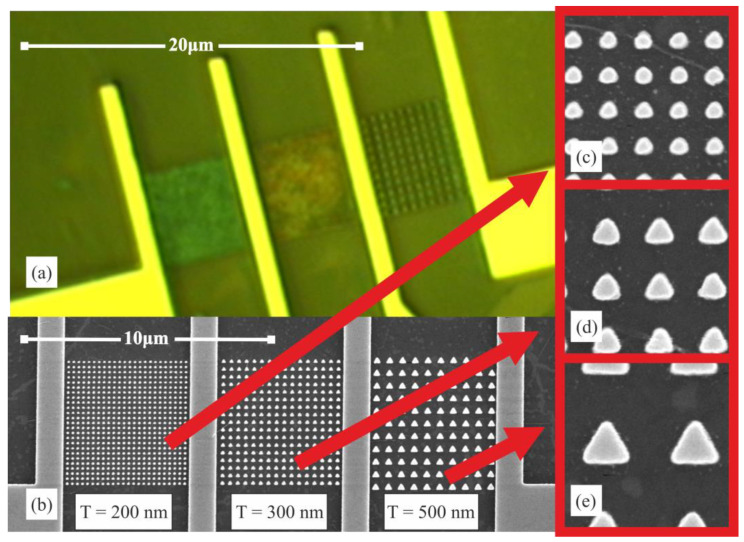
Image of the created photodetectors: (**a**) optical image, (**b**) SEM image of the overall structure, (**c**–**e**) SEM images of nanoprisms with periods T = 200, 300, and 500 nm, respectively.

The contact pads with the electrodes were created in gold with a titanium sublayer. Gold is used because it is not vulnerable to oxidation, has low contact resistance, and is more resistant to mechanical damage. The photosensitive region with plasmonic structures on the semiconductor film is located in the gaps between the electrodes, providing the application of the electric field to the film. Contact pads were provided for probe measurements. Figure 2 shows images of the created plasmonic structures.

As will be shown below, the highest optical absorption occurs only when the plasmonic structures are sufficiently close to each other, at which point the most effective plasmon resonance occurs. However, fabricating the closely spaced plasmonic structures comprises significant technological difficulties. The constraints come from the electron-beam lithography process parameters, such as resist thickness in accordance with feature height and lateral size, density of the pattern, etc. For example, for making a very small gaps (about 100 nm and less) lift-off pattern by means of convenient EBL it is necessary to have rather thin high aspect ratio resist walls (lamellas) in order to provide a reliable lift-off process. On the other hand, such thin resist lamellas are hard to stay in place—they tend to collapse or delaminate, especially when they are based on the poor adhesion sublayer like TMD monolayer, even when the test runs on the bare Si substrate have shown perfect results.

To estimate the created photodetectors’ photosensitivity and to study their polarization sensitivity, the standard method of photocurrent registration was used. The laser beam was focused on the surface of the semiconductor film, to which a constant voltage was applied through tungsten probes connected to the contact pads. The photocurrent value was read using a picoammeter. Changing the polarization of laser radiation was achieved by rotating the half-wave plate. Laser radiation with a wavelength of 635 nm was focused onto a spot with a diameter of 4 μm on the surface of the sample between the electrode structures. Such a large beam diameter is necessary to provide simultaneous illumination of several periods of plasmonic structures. The power density on the sample surface was 550 W/cm^2^.

The geometrical parameters of such low-dimensional structures significantly affect the final characteristics of the created devices [24]. At the same time, their influence can be highly nonlinear. Therefore, in order to evaluate the photodetector efficiency and determine the influence of the plasmonic structure parameters on the photodetector properties, we performed additional physical simulations of optical absorption in a two-dimensional WSe_2_ film.

The simulation consisted of creating an exact copy of the device considered in this work to estimate the distribution of electromagnetic fields in its structure. The model was composed of a silicon substrate, a 90 nm thick oxide layer, a 0.86 nm thick WSe_2_ monolayer film [25], and a 100 nm thick silver plasmonic structure in the form of triangular prisms placed on top of each other. Only one of the structure’s elementary cells, which included one plasmonic element, was simulated. As can be seen from the images shown in Figure 2, the created structures are not geometrically ideal, which was taken into account during modeling.

Since the photocurrent of such detectors depends linearly on the optical absorption in the two-dimensional semiconductor layer, the optical absorption is a determinant of its photosensitivity. The most important results of the calculations were optical amplification (the ratio of local power density to the power density of the radiation incident on the structure), optical absorption in the semiconductor film, and polarization-dependent absorption (the ratio of absorption in the semiconductor film for two mutually perpendicular polarizations of incident light). Optical amplification makes it possible to locally visualize the patterns of LSPR occurrence, and polarization-dependent absorption makes it possible to estimate the sensitivity of the created photodetectors to optical polarization.

## 3. Results

Obviously, these materials possess the maximum optical absorption near their bandgap. However, this does not mean that the plasmonic structures have the greatest polarization anisotropy at the same wavelengths. Therefore, it was necessary to select the optimal wavelengths of optical radiation incident on the created structures. For this purpose, we simulated optical absorption in the created photodetectors at different wavelengths and the azimuthal angle of the incident optical radiation polarization.

**Figure 3:**
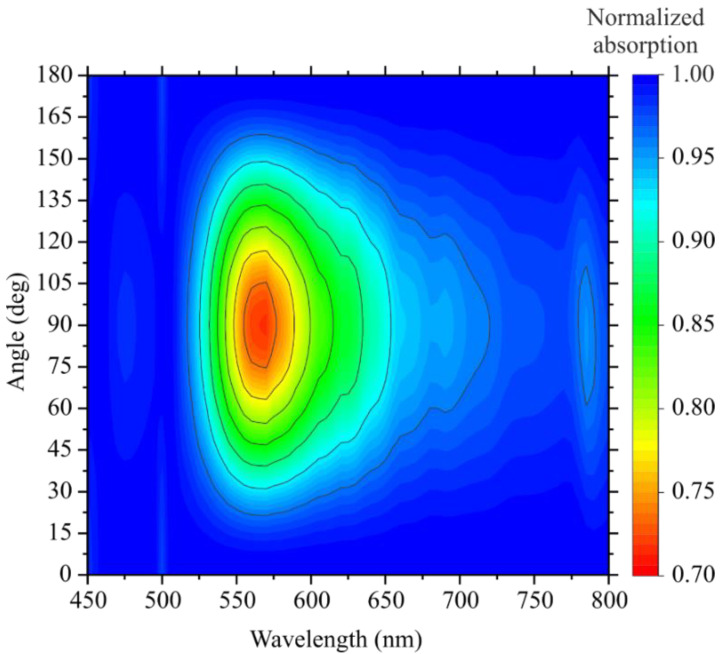
Calculated polarization-dependent absorption in the created photodetector (T = 500 nm) for different values of wavelength and azimuthal angle of the incident optical radiation polarization.

The results of this simulation for the plasmonic structure with period T = 500 nm are shown in Figure 3. For greater clarity, the optical absorption was normalized separately for each value of the incident wavelength, which allows a more detailed evaluation of the anisotropy contribution for each wavelength. The non-normalized raw data are presented in Supplementary Materials S3 (they in fact exactly repeat the absorption value for the WSe_2_ monolayer film).

Thus, the results show that the polarization selectivity (for the T = 500 structure) appears only in a small wavelength range of 550–660 nm. In this case, for optical applications, it is most optimal to use pump wavelengths located as closely as possible to the value of the band gap width of the used semiconductor. Other created structures (with periods of T = 200 and 300) obtained results similar in the form of dependences, but smaller in magnitude (Supplementary Materials S3). Of the most common sources of laser radiation, the dpss lasers with a wavelength of λ = 635 nm are the most closely matched to the indicated wavelengths. Therefore, this wavelength of excitation radiation was used for the experimental analysis of the properties of the created photodetectors.

**Figure 4:**
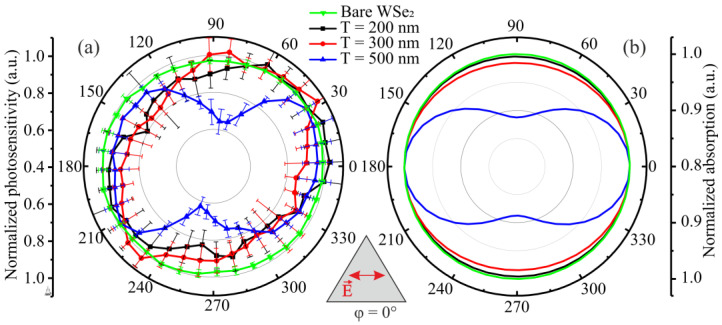
(**a**) Experimentally obtained values of photosensitivity at different polarization angles of incident radiation for photodetectors with plasmonic nanoprisms and (**b**) theoretically calculated absorption in WSe_2_ monolayer with plasmonic structures on its surface at incident radiation with wavelength λ = 635 nm. The inset below schematically shows the polarization direction at angle φ = 0°.

To confirm the obtained theoretical data, the photosensitivity of the created photodetectors was experimentally measured. Due to the large difference in absolute values for each of the plasmonic structures, the normalized photosensitivity is shown in Figure 4a. The actual photosensitivities range from 1.87 to 1.58 uA/W for photodetectors with period structures of 200 nm, 1.50 to 1.29 uA/W for period 300 nm, and 1.31 to 0.85 uA/W for period 500 nm. The photosensitivity of the semiconductor without plasmonic structures was 0.39 uA/W. It is worth noting that such a low photosensitivity is due to the high-power density of the incident radiation, as, with its increase, the value of the photoresponse drops exponentially [26]. Thus, plasmonic structures have allowed the photosensitivity of the created photodetectors to increase considerably. For photodetectors with the period of structures of 200 nm, photosensitivity was increased by a factor of 4.8.

Figure 4b shows the theoretically calculated normalized values of optical absorption in 2D WSe_2_ film with plasmonic structures on its surface obtained at the incident wavelength of 635 nm.

From the obtained theoretical and experimental results, it clearly follows that polarization selectivity at the selected wavelength occurs only for triangular nanoprisms with the period T = 500 nm. Therefore, theoretically, the ratio of optical absorption can reach 1.13 times, while the experimental value of photosensitivity is much higher, 1.6 times. For plasmonic structures with a different period, no polarization selectivity was found.

It can be seen that the absolute values for the experimental and theoretical data are different. It should be noted that the optical coefficients used in the simulation may vary slightly from the real values, which can lead to distortions of the theoretical data. At the same time, the creation of plasmonic structures on the semiconductor film surface should not change its own optical coefficients. This conclusion can be drawn because the gentlest techniques for creating a metal layer on the TMD surface are used. Thermal vapor deposition is considerably less degrading than magnetron or laser sputtering. At the same time, silver is not a refractory material and requires a relatively low temperature for its evaporation. It should also be noted that the WSe_2_ film used is quite inert and does not interact with the metal. Therefore, we can assume that the metal sputtering itself does not greatly affect the change in the optical parameters of the film. However, it is not possible to measure this directly in principle.

Moreover, the theoretical models created may differ somewhat from their real embodiments. Thus, we cannot reliably determine the homogeneity of plasmonic structures’ vertical walls or accurately estimate the geometry of their faces. For example, they may have the shape of an inclined parallelepiped. We have analyzed all their geometric features, but there will still definitely remain discrepancies, which of course will lead to differences between theoretical and experimental results.

The obtained theoretical and experimental data for plasmonic structures with a period of T = 500 nm indicate a 180-degree periodicity of polarization-dependent absorption in a semiconductor film on the azimuthal angle of incidence of laser radiation polarization. In this case, the phases of the azimuthal dependences also coincide. The maximum corresponds to the case when the polarization is parallel to the base of the triangle φ = 0° (the polarization direction is indicated in the inset below in Figure 4).

**Figure 5:**
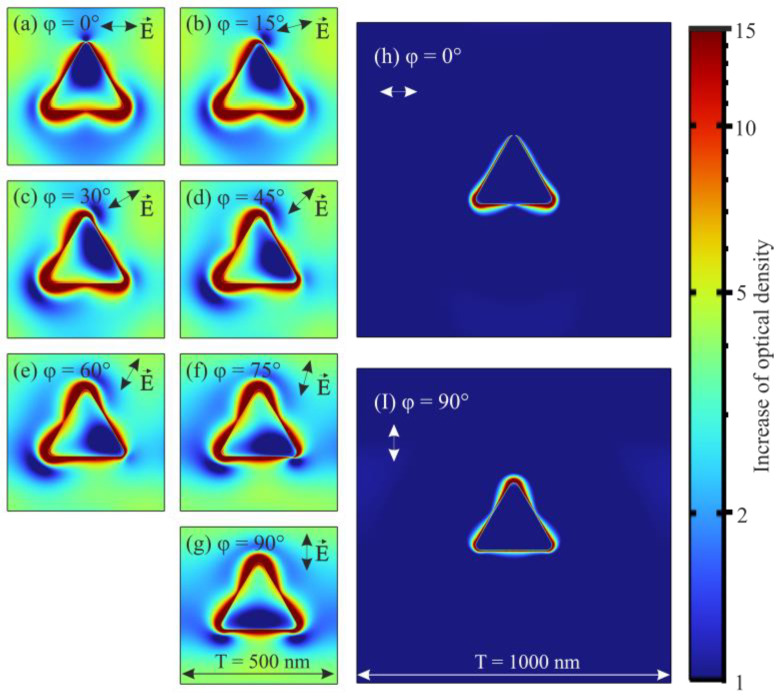
Calculated optical radiation amplification distribution in the layer of 2D WSe_2_ films for plasmonic structures at the incident radiation wavelength λ = 635 nm (**a**–**g**) for the created photodetectors with period T = 500 nm, (**h**,**i**) for photodetectors with doubled spacing between them.

In order to analyze the reason for this regularity, a simulation of the local amplification of optical radiation in the semiconductor layer was performed. The results of the simulation (the cross section is conducted in the middle of the semiconductor volume) for different incident polarizations of optical radiation in the calculated unit cell with the period of plasmonic structures T = 500 nm are shown in Figure 5a–g. It can be noted that, near the metallic plasmonic structures, there is the greatest increase in the power density of the optical radiation, the distribution of which strongly depends on the polarization parameters. When the incident polarization rotates, the greatest amplification is observed at the corners of the triangle, which are adjacent to the base parallel to the polarization of the incident light. However, such local amplification occupies a relatively small surface area, and therefore it is not its local peak value that is important, but the average value of amplification over the whole area of the semiconductor.

The results show that the amplification of optical radiation occurs on the entire surface of the semiconductor, which is located in the calculated unit cell. For example, for the polarization incidence angle φ = 0°, the amplification to the left and right of the triangular prism increases fivefold and to the top and bottom is close to unity. As the polarization rotates, such a symmetrical field distribution pattern also begins to rotate following the polarization (Figure 5a–g). Such amplification of optical radiation so far away (more than the wavelength of incident radiation) from the plasmonic structure can be explained by interaction between plasmonic structures, leading to SPR.

To verify this assertion, a simulation of optical radiation amplification was performed for a triangular nanoprism of the same size but with twice the distance between them. The results of this simulation are shown in Figure 5h,i. They show that the amplification is located only in a very small region in the immediate vicinity of the plasmonic structures and does not spread farther. This field distribution is characteristic of the LSPR. It was found that, for such modified structures, polarization anisotropy is completely absent (see Supplementary Materials S4), which fully confirms the occurrence of polarization selectivity due to the interaction between the plasmonic structures and not due to their anisotropic shape. Moreover, single triangular prisms, by virtue of their shape, should possess 60-degree symmetry of polarization dependences, which also does not correspond to the obtained results. In addition, it was found that the polarization selectivity of nanoprisms can be altered by rotating the prisms within the ordered matrix in which they are placed (see Supplementary Materials S5). The maximum value of polarization-dependent absorption is observed when one of the sides of the triangle is parallel to the axes of the square matrix in which they are ordered. It has been demonstrated that it is possible to completely get rid of polarization-dependent absorption by rotating the triangular nanoprisms by 15 degrees.

**Figure 6:**
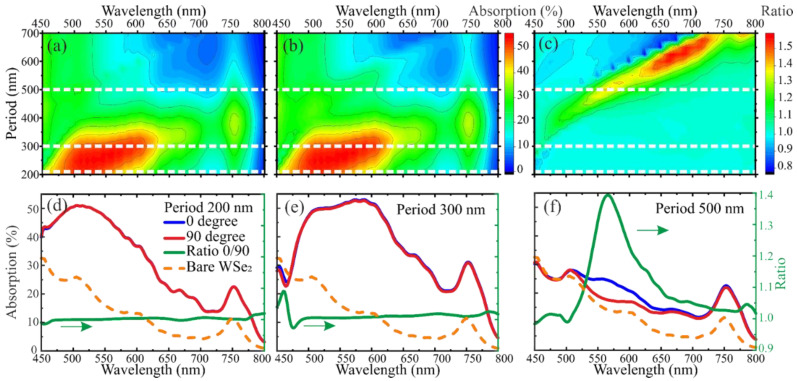
Simulation results of the optical absorption dependence in a two-dimensional WSe_2_ film on the plasmonic structure period and the incident radiation wavelength with (**a**) φ = 0° and (**b**) φ = 90°. (**c**) The absorption ratio at φ = 0° to φ = 90°. Dependence of optical absorption for different azimuthal angles of incident polarization at (**d**) T = 200 nm, (**e**) T = 300 nm, and (**f**) T = 500 nm.

Thus, if it is known that the maximum ratio of optical selectivity in such structures is achieved at azimuthal angles of incidence of laser radiation φ = 0° and φ = 90°, then it is possible to estimate the maximum possible polarization selectivity for such structures. Thus, Figure 6a,b shows the calculated absorption in the WSe_2_ monolayer based on this model as a function of wavelength and period of plasmonic nanoprisms for polarization angles φ = 0° and φ = 90°, respectively. Figure 6c shows the polarization absorption ratio for this structure, which was obtained by dividing the absorption values at polarization angles φ = 0° and φ = 90° by each other. The results show that by changing the period of the structures, the polarization selectivity can be achieved over the entire visible optical range. Moreover, as can be seen from the presented results, such a change also leads to the appearance of spectral absorption selectivity. Based on the obtained results, the maximum polarization selectivity can be obtained using triangular nanoprisms with T = 600 and an incident wavelength of 675 nm. Theoretically, the accurate selection of parameters of plasmonic structures and pump wavelengths will increase the polarization selectivity at least an additional five times for the created photodetectors.

Additionally, Figure 6d–f shows absorption spectra for plasmonic structures with the same periods T = 200, 300, and 500 as for the created photodetectors. They show values of optical absorption for different values of azimuthal angles of incident polarization of laser radiation and their ratio. For comparison, all graphs additionally show the spectral absorption in the semiconductor film without a plasma structure deposited on its surface. Thus, it can be seen that the maximum value of anisotropy does not coincide with the value of the bandgap width of the semiconductor. For triangular nanoprisms with a period of T = 200 and T = 300, polarization absorption anisotropy is absent over the entire visible wavelength range. For triangular nanoprisms with period T = 500, significant anisotropy is observed at a wavelength of about 560 nm.

The ultraviolet wavelength range up to 200 nm was also investigated using theoretical simulations. The results are presented in Supplementary Materials S6. In the ultraviolet range, the absorption in the TMD film is not high. This effect is due to changes in light interference conditions in the TMD/SiO2/Si structure. It is also determined that the absorption anisotropy in the ultraviolet region has a rather small value not exceeding 1.15 (versus 1.4 in the visible range). Moreover, the spectral width of such absorption anisotropy peaks is very narrow, which greatly complicates their possible use.

## 4. Conclusions

In summary, in this work, polarization-sensitive photodetectors based on two-dimensional semiconductor films that initially do not have polarization sensitivity were developed. For this, arrays of ordered asymmetric silver nanoprisms were used, due to which a surface plasmon resonance appeared in a semiconductor film. It was experimentally shown that the polarization selectivity (the maximum ratio of photosensitivities for mutually perpendicular polarizations of the incident light) of the developed detectors is 1.6 times. It was found that the use of ordered metal structures makes it possible to increase the photosensitivity of the detectors by a factor of five. Using physical modeling, we showed that the efficiency of such structures can be significantly increased over the entire visible spectral range by varying the corresponding form factors of plasmonic structures.

## Data Availability

The data presented in this study are available on request from the corresponding author.

## Supplementary Materials

The following supporting information can be downloaded at: https://www.mdpi.com/article/10.3390/nano12111854/s1, Figure S1: Simulation results of the optical absorption dependence in a two-dimensional WSe_2_ film on the Ag (a–c) and Au (d–f) plasmonic structure period and the incident radiation wavelength with (a,d) φ = 0° and (b,e) φ = 90°. (c,f) The absorption ratio at φ = 0° to φ = 90°; Figure S2: Dependence of the optical radiation absorption in the two-dimensional WSe_2_ film on the thickness of the plasmon element for two values of the polarization rotation angle φ of the incident electromagnetic wave. Form-factor of the plasmon element: triangular nanoprism with period T = 500 nm. The incident radiation wavelength is 635 nm; Figure S3: 3D distribution of the optical gain in the simulated unit cell for different thicknesses of triangular nanoprisms and angle φ: (a–c) Thicknesses of 50, 100, 200 nm for angle φ = 0°. (d–f) Thicknesses of 50, 100, 200 nm for angle φ = 90°. The blue isosurface shows an optical gain of 10, the red one 20; Figure S4: Dependence of optical radiation absorption in two-dimensional WSe_2_ film as a function of the size of plasmonic structures D and their period T. The results were obtained for two values of the incidene light polarization angle (a) φ = 0°, (b) φ = 90°, and (c) absorption ratio. The incident wavelength was 635 nm; Figure S5: Calculated polarization-dependent absorption in the created photodetector for different values of wavelength and azimuthal angle of the incident optical radiation polarization. (a–c) Normalized absorption for T = 200nm, T = 300nm, T = 500nm, respectively. (d–f) Non-normalized absorption for the same periods; Figure S6: Calculated polarization-dependent absorption in WSe_2_ monolayer with double period T = 1000 nm plasmonic structures (see Figure 5h–i) on its surface at incident radiation with wavelength λ = 635 nm; Figure S7: Absorption dependence in two-dimensional WSe_2_ film on the rotation angle Θ of triangular nanoprisms for two values of the polarization rotation angle of the incident electromagnetic wave φ; Figure S8: (a) Absorption spectrum of the structures as a function of plasmon period T and radiation polarization, (b) absorption ratio at φ = 0° and φ = 90°.
