# Supplementary material for: Polarization Sensitive Photodetectors Based on Two-Dimensional WSe2
Source: Nanomaterials (Basel). 2022 May 29;12(11):1854. doi: 10.3390/nano12111854 (PMC9182037; doi:10.3390/nano12111854)
Supplement: Supplementary file 1 [file nanomaterials-12-01854-s001.zip › nanomaterials-1727984-supplementary.pdf]

Supplementary

# Polarization Sensitive Photodetectors Based on Two-Dimensional $WSe_2$

**Andrey Guskov [1], Sergey Lavrov [1,*] and Rinat Galiev [2]**

[1] Department of Nanoelectronics, MIREA—Russian Technological University, Moscow 119454, Russia; guskov@mirea.ru
[2] V.G. Mokerov Institute of Ultra High Frequency Semiconductor Electronics of RAS, Moscow 117105, Russia; rgaliev@isvch.ru
* Correspondence: sdlavrov@mail.ru; Tel.: +7-499-215-65-65 (ext. 3003)

## S1. Selection of material for the plasmonic structure

For a more detailed analysis of the choice of material for the plasmonic element, we also performed additional simulations for plasmonic structures made of Au and Ag. The results of the simulation are shown in Figures S1. If we compare it with Figure S1(a-c) for Ag, we can see that the maximum absorption for Ag is higher for the chosen spectral range. Therefore, at the used wavelength of 635 nm for Ag, plasmonic structures absorption in the semiconductor layer reaches 16%, and only 11% for Au. It is this difference in absorption that dictates the choice of Ag to create plasmonic elements.

However, it should be noted that the obtained value of polarization-selective absorption (absorption ratio for two mutually perpendicular polarizations of incident light polarizations) is almost identical for these materials and is equal to 1.13 and 1.14 for Au and Ag, respectively.

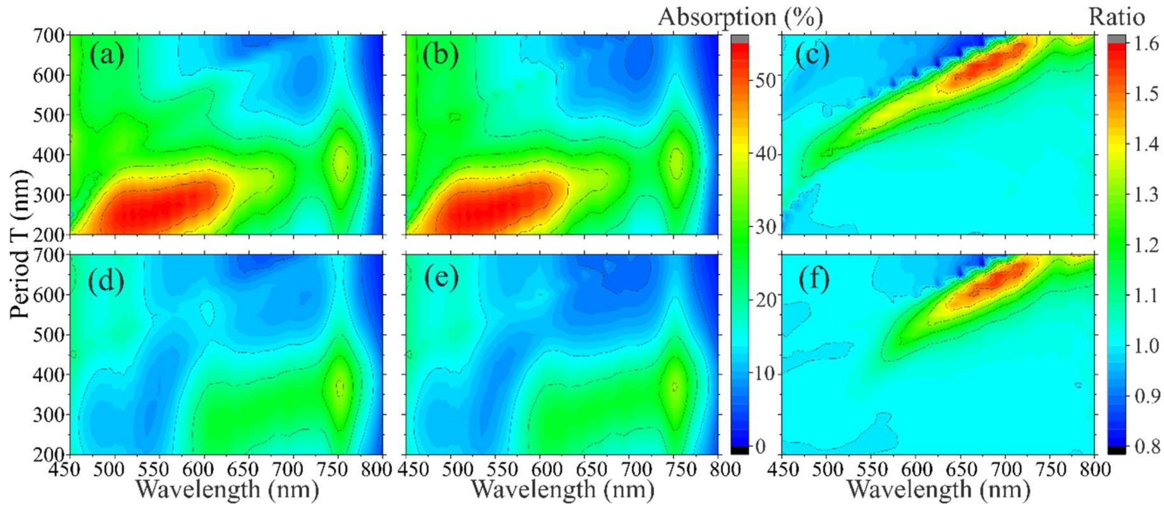


Figure S1. Simulation results of the optical absorption dependence in a two-dimensional $WSe_2$ film on the Ag (a–c) and Au (d–f) plasmonic structure period and the incident radiation wavelength with (a,d) φ = 0° and (b,e) φ = 90°. (c,f) The absorption ratio at φ=0° to φ = 90°.

## S2. Modeling the optimal form-factor of plasmonic structures

The shape of the nanoprisms is selected by means of physical modeling in two steps:

1. Determining the optimal thickness of the ordered plasmonic structures.
2. Determination of the optimal size of the plasmonic structures and the matrix period in which they are ordered.

During the first stage, a theoretical model was built, which made it possible to determine the dependence of absorption in the semiconductor layer depending on the thickness of metal plasmonic elements. The result of such theoretical dependence for the triangular nanoprism with period T=500 nm for wavelength 635 nm considered in the article is presented as an example in Figure S2. The results for two polarization angle values of the incident electromagnetic wave φ are calculated. From the obtained results it can be seen that the maximum value of optical absorption does not exceed 16% at the plasmonic structure thickness of about 125 nm for the value φ=0°.

However, as follows from the presented results, small metal thicknesses are optimal for polarization selectivity. Thus, Figure S2 shows that the optimal thickness is 30 nm. Smaller values are not presented in the graph as in this case the plasmonic structures become too thin and actually become transparent to the incident electromagnetic radiation. In addition, such films are technologically more difficult to make, they are less stable and can easily be destroyed when irradiated by a laser source or contact probes. Therefore, in this case, the optimum thickness of the plasmonic structures was chosen to be 100 nm. It is worth noting that a similar dependence holds for all values of the periods of the plasmonic structures T.

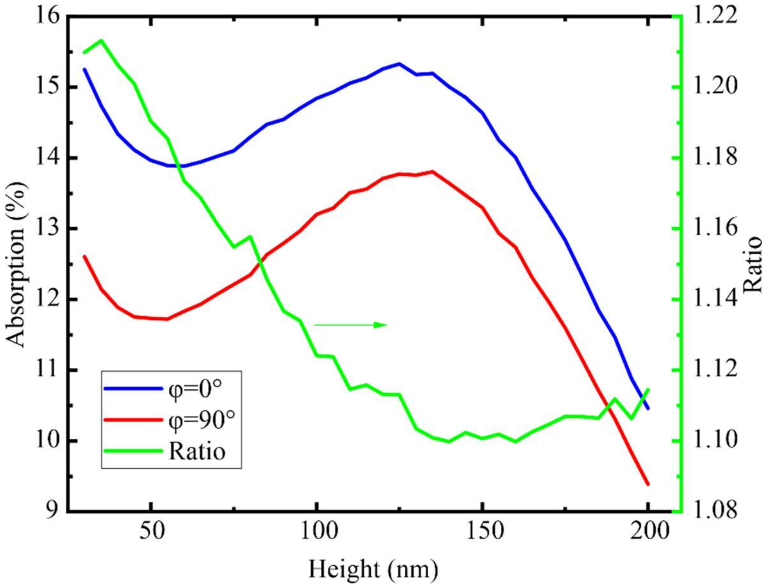


Figure S2. Dependence of the optical radiation absorption in the two-dimensional $WSe_2$ film on the thickness of the plasmon element for two values of the polarization rotation angle φ of the incident electromagnetic wave. Form-factor of the plasmon element: triangular nanoprism with period T=500 nm. The incident radiation wavelength is 635 nm.

The dependence shown in Figure S2 can be explained as follows: local amplification of the electromagnetic field for triangular nanoprisms occurs on their lower and upper faces when radiation is incident from above. Thus it can be considered that these faces are the main sources of local plasmon resonance. If the thickness of the triangular prism is significant (more than 200 nm), the upper face will not contribute to the amplification of the optical radiation power density in the two-dimensional semiconductor film, as it will be located far from it. Moreover, the greater thickness of the metal will reduce the intensity of the optical radiation hitting the lower face of the nanoprism located directly on the semiconductor. This is why the optical absorption drops as the thickness of the metal layer increases. However, when the metal layer is significantly reduced, the plasmon resonance sources begin to overlap and the power density also decreases slightly. Images of the optical gain distribution (local increase in the incident radiation intensity) for the thicknesses of plasmonic structures of 50, 100, and 200 nm at different values of the azimuthal angle φ are shown in Figure S3.

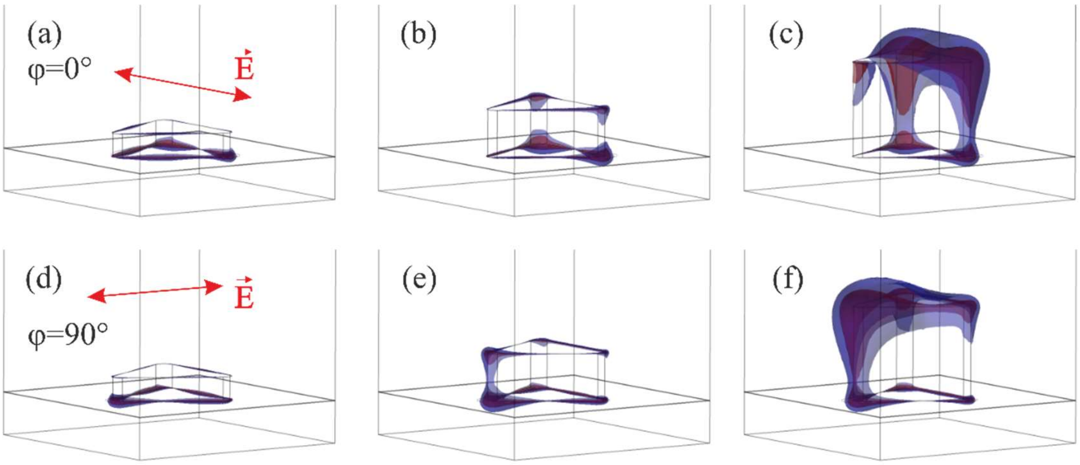


Figure S3. 3D distribution of the optical gain in the simulated unit cell for different thicknesses of triangular nanoprisms and angle φ: (a–c) Thicknesses of 50, 100, 200 nm for angle φ=0°. (d–f) Thicknesses of 50, 100, 200 nm for angle φ=90°. The blue isosurface shows an optical gain of 10, the red one 20.

At the second stage of modeling the optimal dimensional characteristics of the plasmonic structures were determined. Thus, the dependences of absorption in a two-dimensional semiconductor on the size of triangular nanoprisms and the period of the square matrix in which they are ordered were constructed. In this case, the characteristic size of the plasmon is the diameter of the circumscribed triangular circle (hereafter denoted as D), which makes up the upper face of the nanoprism. The period of the plasmonic structures (the distance between their centers) is denoted as T. Thus, if D will be greater than T, then depending on the angle of rotation of the plasmon element, the plasmon elements may intersect. The results of this simulation are shown in Figure S4.

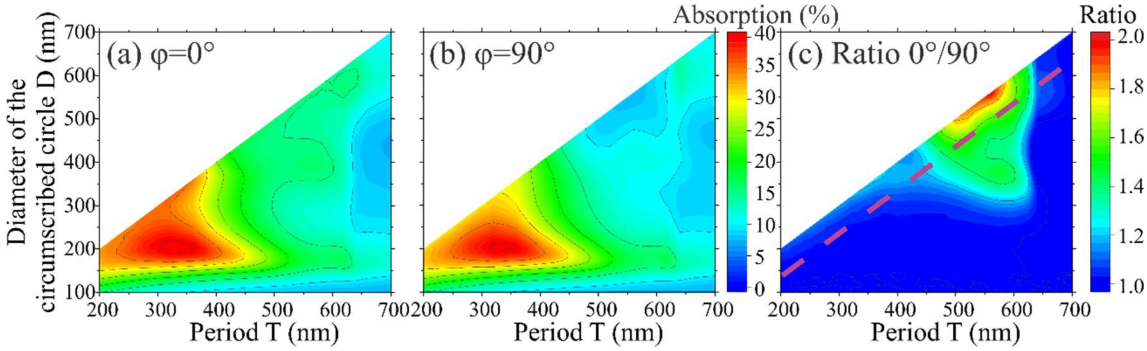


Figure S4. Dependence of optical radiation absorption in two-dimensional $WSe_2$ film as a function of the size of plasmonic structures D and their period T. The results were obtained for two values of the incidene light polarization angle (a) φ=0°, (b) φ= 90°, and (c) absorption ratio. The incident wavelength was 635 nm.

As can be seen from Figures S4(a) and S2.3(b), for both polarizations φ=0° and φ=90° the absorption map has a similar appearance. Thus, they have an absorption maximum of 40% at period T=340 and diameter D=190 nm. Taking into account that the plasmon-free film at this wavelength absorbs only about 10% (see Figure 6 in the article), this chosen form factor of plasmonic metal structures gives a significant efficiency gain. However, the absorption ratio (Figure S4(c)) for this maximum point gives a value equal to one, which makes such a form factor of plasmonic structures unsuitable for polarization-selective applications. However, Figure S4(c) traces a region where the absorption ratio can reach the value of two. It is this region that is of most interest for polarization-sensitive applications.

However, the fact that we wanted to make the coverage area of the semiconductor surface with metallic plasmonic structures constant is extremely important. In this case, we excluded the possibility of changing the efficiency of the photodetector only due to the fact that the area of the working surface (the

regions of the semiconductor film open to optical radiation) of the photodetector changed. Accordingly, for this reason, in our work, when changing the period of plasmonic structures, the size of the triangular prism also changed. In our case, the area under the plasmonic structures was fixed and was approximately 15%.

It is worth noting that if the D value is close to T, it leads to a close arrangement of metal elements. Since the elements are quite thick, such a structure is technologically difficult to fabricate. Therefore, it was necessary to deviate slightly from the optimal values to ensure sufficient defect-free structures. The technological problems of creating closely spaced elements are described in more detail below.

Therefore, a D/T ratio of 0.7 was chosen when creating the plasmonic structures. The selected ratio is shown in Figure S4(c) as a dashed line.

### S3. Spectral absorption modeling

For all types of plasmonic structures, maps were obtained of the absorption dependence in a two-dimensional semiconductor on the wavelength of incident light and the azimuthal angle of its polarization. Figures S5(a–c) show the wavelength-normalized absorption values. Figures S5(a–c) show the absorption values. It follows from the presented results that for the periods T=200 and T=300 the maximum difference in absorption for different polarizations does not exceed 5%, while for T=500 the difference is more than 30%.

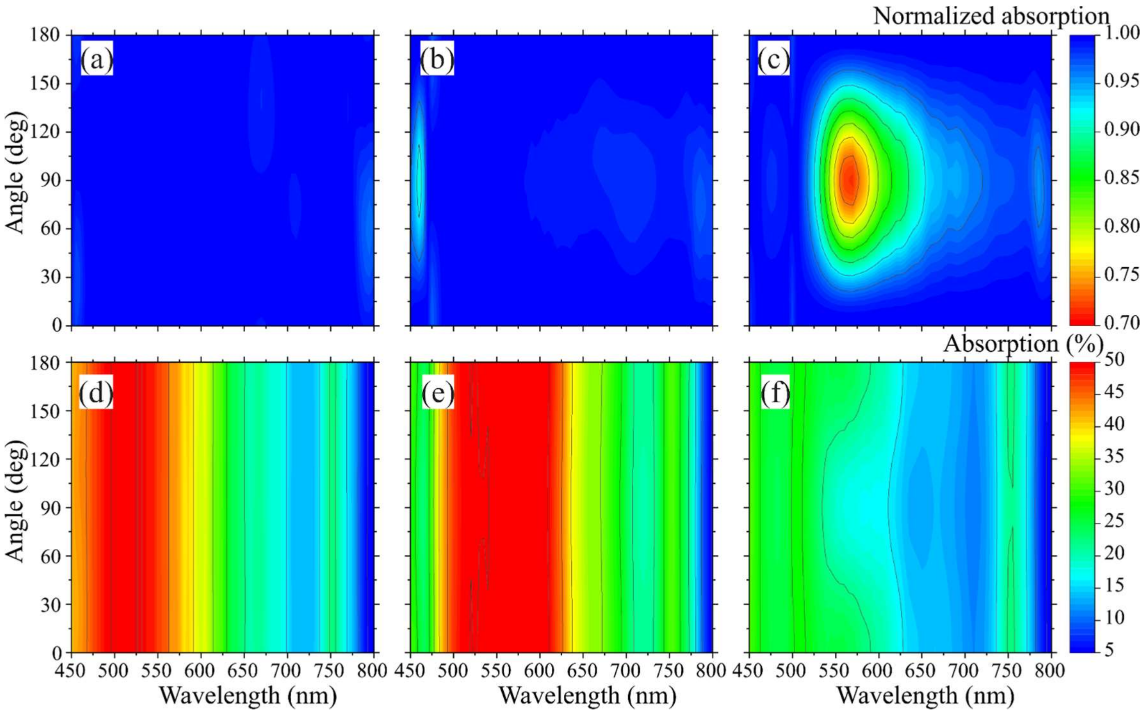


Figure S5. Calculated polarization-dependent absorption in the created photodetector for different values of wavelength and azimuthal angle of the incident optical radiation polarization. (a–c) Normalized absorption for T=200nm, T=300nm, T=500nm, respectively. (d–f) Non-normalized absorption for the same periods.

### S4. Absorption simulation for a structure with doubled distance between plasmonic elements

To verify the presence of polarization-selective absorption in the $WSe_2$ film in the case where there is no interaction between plasmons, a simulation of the structure with a doubled distance between plasmons was performed. The absorption dependence in the $WSe_2$ film on the incident radiation polarization azimuthal angle is shown in Figure S6. It shows that the difference in absorption does not exceed 1%.

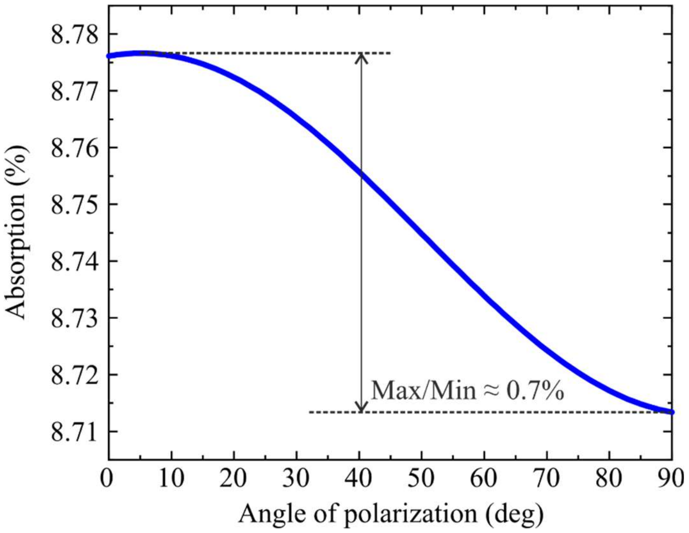

Figure S6. Calculated polarization-dependent absorption in $WSe_2$ monolayer with double period T=1000 nm plasmonic structures (see Figure 5 (h–i)) on its surface at incident radiation with wavelength $\lambda$=635 nm.

**S5. Nanoprisms orientation modeling**

For the triangular nanoprisms considered in the project the orientation of the structures plays a decisive role in their polarization selectivity. In spite of the fact that periodicity for rotation of polarization of the incident radiation in this work is indicated by 180 degrees, it is different for rotation of the plasmonic nanoprisms themselves. Thus, Figure S7 shows the simulation of optical absorption in the semiconductor layer as a function of the rotation angle Θ of the triangular nanoprisms. The dependence is periodic with a period of 60 degrees. It can be seen that for orthogonal polarizations of electromagnetic waves φ=0° and φ=90° the dependences are shifted relative to each other by half a period. We can also conclude that when plasmonic nanoprisms are rotated by 30 degrees, they completely lose their polarization-sensitive properties.

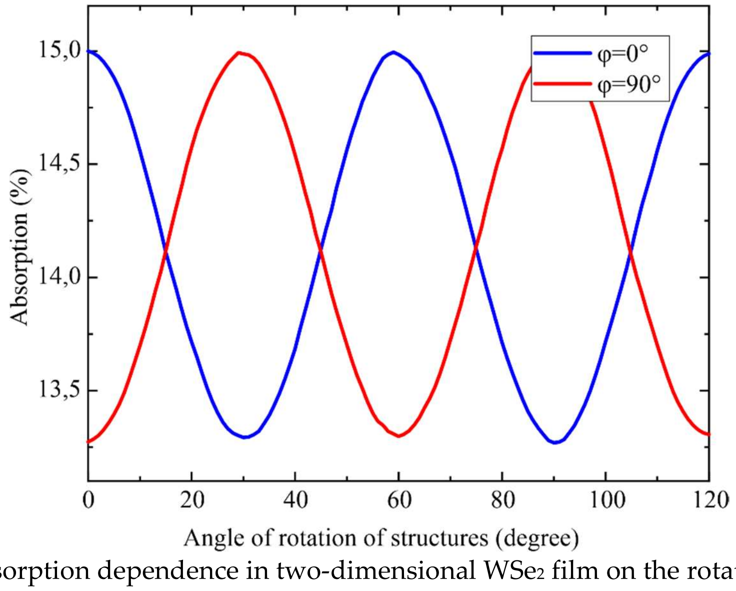

Figure S7. Absorption dependence in two-dimensional $WSe_2$ film on the rotation angle Θ of triangular nanoprisms for two values of the polarization rotation angle of the incident electromagnetic wave φ.

At the same time, as was shown earlier in the supplementary material to the article, individual plasmonic structures themselves do not possess such optical anisotropy. From this we can conclude that

such dependence arises only due to the redistribution of the local plasmonic gain during the interaction between the nearest, neighboring, metal structures.

It is worth noting that for the case of a square ordering matrix of plasmonic elements, only plasmonic structures with odd-order axial symmetry have such a feature. For structures with nanodisc or nanosquare form factors such dependence is absent.

## S6. Optical absorption in the broad spectral range

Additional theoretical modeling of optical absorption in the semiconductor layer was performed to evaluate the efficiency of the developed photodetectors in the ultraviolet optical range. As indicated earlier, the optical constants for the $WSe_2$ semiconductor monolayer film were taken from [Jung G. H., Nanophotonics, 2019]. Unfortunately, the authors of this paper provide constants only for the visible wavelength range, so it is impossible to perform absorption simulations in the UV spectrum using these data. We were only able to find one noteworthy source [Gu H., Nanoscale, 2019] that estimates shorter wavelengths for this material. It is worth noting that the optical parameters for the two-dimensional film in both of the above sources are qualitatively correlated with each other, but quantitatively the data differ slightly. These distinctions, of course, lead to different results of conducted modeling, but the general tendency of all dependencies remains unchanged.

Below are graphs of absorption dependence on the incident radiation wavelength (UV and visible spectrum) for different periods of plasmonic structures (Figure S8(a)) and absorption ratio for two mutually perpendicular polarizations of the incident radiation (Figure S8(b)).

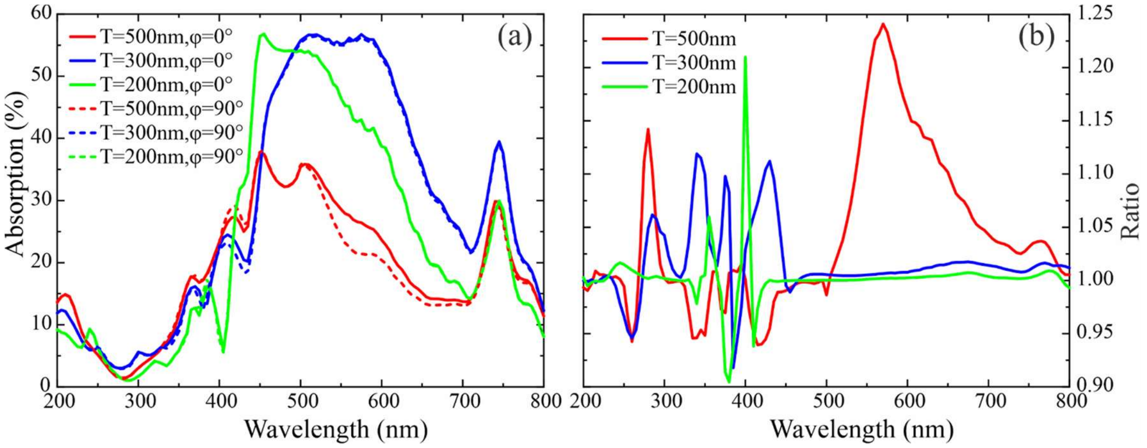


Figure S8. (a) Absorption spectrum of the structures as a function of plasmon period T and radiation polarization , (b) absorption ratio at φ=0° and φ=90°.

From the obtained results it can be concluded that the absorption in the UV spectrum is very small and amounts to no more than 15%, which is shown in Figure S8(a). Figure S8(b) shows that the high values of absorption anisotropy in the two-dimensional semiconductor film are very "narrow" in the UV range, which makes them very difficult to use. At the same time, we see a wide maximum of the absorption ratio in the visible spectrum (exactly the same dependences but with slightly larger amplitudes are presented in our paper in Figure 6(d–f)).
